# Exploring Stakeholders’ Perceptions of Using Digital Health Technologies to Improve the Conservative Treatment of Adolescent Idiopathic Scoliosis: Qualitative Study

**DOI:** 10.2196/69089

**Published:** 2025-06-25

**Authors:** Kamila Sykorova, Anna Mathew, Nenad Pavel, Parisa Gazerani, Trust Saidi, Marianne Bakke Johnsen, June Ullevoldsæter Lystad, Helen Bull, Mette Fløystad Kvammen, Hong-Gu He, Julia Jacoby, Minna Pikkarainen

**Affiliations:** 1 Department of Rehabilitation Science and Health Technology Faculty of Health Sciences OsloMet – Oslo Metropolitan University Oslo Norway; 2 Department of Product Design Faculty of Technology, Art and Design OsloMet – Oslo Metropolitan University Oslo Norway; 3 Department of Life Sciences and Health Faculty of Health Sciences OsloMet – Oslo Metropolitan University Oslo Norway; 4 Department of Psychology University of Oslo Oslo Norway; 5 Division of Mental Health and Addiction University of Oslo Oslo Norway; 6 Department of Sociology and Social Work University of Agder Kristiansand Norway; 7 Norwegian Spine and Back Pain Association Oslo Norway; 8 Alice Lee Centre for Nursing Studies Yong Loo Lin School of Medicine National University of Singapore Singapore Singapore

**Keywords:** adherence, braces, digital health, focus groups, mobile app, mobile health, monitoring, peer support, telemedicine, scoliosis

## Abstract

**Background:**

Adolescent idiopathic scoliosis (AIS) affects 2%-3% of adolescents, with conservative treatments like bracing and physiotherapeutic scoliosis-specific exercises (PSSEs) recommended for mild to moderate cases. However, patient compliance with these treatments is often low. Digital tools, including smartphone apps and web applications, offer capabilities such as spinal curvature monitoring, remote consultation, and reduction of health care professionals’ workload. These tools may also enhance adherence by increasing motivation and providing real-time feedback, which can be particularly beneficial for patients struggling with brace discomfort or self-esteem concerns. Despite these potential benefits, research remains limited on how digital health technologies can specifically enhance conservative AIS treatment and improve patient outcomes.

**Objective:**

This study aimed to explore the perspectives of multiple stakeholders, including patients, caregivers, and health care providers, on using digital health technologies to improve AIS treatment adherence and outcomes.

**Methods:**

This qualitative research study was conducted in Norway and included 17 participants (1 medical doctor, 2 physiotherapists, 8 patients, 4 family caregivers, and 2 IT specialists). The study adhered to Norwegian regulations. After approval from authorities and approval of the study protocol, patients were recruited through the Norwegian Spine and Back Pain Organization. A portion of the sample was recruited through direct communication from one of the researchers. After obtaining written informed consent from all participants, 5 focus group interviews were conducted between April and June 2023. Data were transcribed, translated, and analyzed using a content analysis approach, with findings reviewed by 2 independent researchers for validation.

**Results:**

The content analysis revealed four key themes: (1) AIS-specific education and information, (2) psychosocial support for patients with AIS and community connection, (3) health care communication and access, and (4) treatment adherence to AIS and gamification. Participants highlighted the need for accessible, adolescent-friendly, and multilingual education on AIS; digital platforms for peer support; improved remote communication with health care providers; and gamification elements tailored to AIS challenges (eg, brace compliance tracking, avatar customization for self-expression, and real-time exercise feedback).

**Conclusions:**

Key findings highlighted the need for accessible information, peer support, and better communication with health care providers, with gamification enhancing treatment adherence. The findings of this study show the potential of digital health technologies in AIS management through fostering accessible information, peer support, and improved communication with health care providers. Customized gamification features may further enhance adherence, offering actionable insights for clinical practice and future research.

## Introduction

### Background

Scoliosis involves 3D curvature of the spine [1]. Adolescent idiopathic scoliosis (AIS) affects 2%-3% of adolescents (aged 10 years to maturity), with a higher prevalence among females [2,3]. Treatment strategies vary based on risk progression, bone maturity, and the current stage of spinal curvature [4,5].

### Current AIS Treatment

AIS is treated either surgically or conservatively. Surgical intervention is considered for curves between 40 and 50 degrees [4], while conservative brace treatment is recommended for growing children with curves between 25 and 40 degrees. Scoliosis-related surgeries often involve high levels of pain and slow return of function, with risks including infection, neurological injury, and pulmonary and respiratory complications [6]. Additionally, surgery is associated with chronic anxiety or depression [7]. Enhanced recovery protocols promote early mobilization to shorten hospital stay, but the average length of stay varies [8,9].

Conservative treatment is often recommended for children or young people with mild to moderate deformities [10]. This includes bracing and physiotherapeutic scoliosis-specific exercises (PSSEs) for patients with Cobb angles between 20 and 40 degrees [11]. Scoliosis-specific exercise therapy and bracing have been shown to improve long-term outcomes and quality of life [12,13]. The success of brace treatment is directly related to the number of hours the brace is worn [14]. Patients recommended for brace treatment typically have spinal curves between 30 and 40 degrees or 20 and 29 degrees with rapid progression [15]. The goal of bracing combined with PSSEs is to postpone or avoid surgery [16,17].

While conservative treatment is theoretically effective, practical challenges exist in conservative treatment adherence, primarily due to discomfort, pain, irritation, sweating, and self-esteem issues [15]. High demand for movement correction often requires constant physiotherapy supervision, which is not always accessible at home. More feedback results in more precise exercising, especially in children [18]. As smartphone use involving digital technology among adolescents continues to rise, innovative technologies, particularly smartphone health apps, present new opportunities to support and enhance treatment compliance. These apps can monitor symptoms, collect real-time data, and provide personalized feedback, making them a promising tool for improving self-management and brace-wearing compliance in patients with AIS. Given the growing need for effective, user-friendly solutions in scoliosis care, the integration of such technologies could not only improve clinical outcomes but also empower patients to take a more active role in managing their condition [19].

### Digital Health Technologies in AIS Treatment

Telehealth and telemonitoring are gaining importance in the health sector [20]. There is potential to use digital health technologies, such as smartphone apps and web applications, in AIS treatment with existing technologies. These digital health technologies could bring several advantages to both patients and health professionals [19,21]. The current literature primarily focuses on digital health technologies for scoliosis screening [22]. Smartphone apps can provide periodic monitoring of spinal curvature progression, reducing the need for frequent in-person visits and offering quick consultations and feedback on exercises [21]. Digital health technologies can promote self-management and strengthen patient involvement in monitoring their health conditions [23].

In this context, digital health technologies present new possibilities to address these treatment barriers, as they enable clinicians to oversee a greater number of scoliosis patients while delivering immediate feedback based on exercise outcomes. Management based on information and communication technologies simplifies interprofessional communication and information exchange securely, enabling remote communication of health issues and overcoming logistical challenges. Apps can help doctors organize and track appointments, meetings, and administrative routines [24]. Digital health technologies promise to reduce health care professionals’ workload and improve patient self-management [25]. The use of technologies needs to be properly considered regarding ethics, legislation, fairness, and privacy matters, especially in the medical field [26].

Digital tools can encourage early self-screening and detection of scoliosis, preventing complications. A scoliosis screening system using standard 2D digital cameras or smartphone sensors can facilitate global detection efforts. Measurements using apps are more stable and precise due to sensors like accelerometers and gyroscopes. Smartphone apps and web applications are appropriate for monitoring and supervision in home-based treatment programs [21].

Self-monitoring can motivate patients in their treatment journey, offering empowerment, education, and an accessible platform for communication between clinic visits. Monitoring apps can connect patients with health personnel to address discomfort, answer treatment questions, and provide immediate feedback [15]. Retrospective reports at doctors’ offices are problematic as they do not allow early detection of issues. In these cases, it is important for the patient to talk about the discomfort, which could potentially lead to more continuous brace use until the next appointment [15].

There is a notable research gap in conservative AIS treatment. While some studies describe digital tools in surgical scoliosis treatment [21], few empirical studies explain how to use digital health technologies to improve conservative AIS treatment [15]. Furthermore, there is limited understanding of the digital health functionalities and features required by patients, their caregivers, and therapists. Unlike other chronic conditions, AIS presents specific compliance barriers, such as body image concerns, long-term commitment, pain and discomfort, and health care access limitations. Adolescents may feel self-conscious about wearing braces, leading to low adherence, while the long-term commitment required for bracing and physiotherapy can be discouraging without immediate visible results. Additionally, bracing may cause sweating, pressure pain, and restricted movement, further affecting daily activities. Long intervals between check-ups can also leave patients with limited support, exacerbating their challenges [15,21].

The aim of this qualitative interview study is to explore how digital health technologies can be tailored to address AIS-specific challenges and improve conservative treatment in Norway. The study will also critically assess whether the potential of digital health solutions is meaningful and desirable for users, ensuring that the technologies developed are both effective and aligned with the needs of patients, caregivers, and therapists.

## Methods

### Design Strategy

The qualitative research detailed in this paper was conducted in Norway (2023-2024) and involved 17 participants, including 1 medical doctor, 2 physiotherapists, 8 patients, 4 family caregivers, and 2 IT specialists. The study employed a combination of physical and hybrid participation formats across 5 focus group interviews conducted between April and June 2023.

Focus group interviews were chosen to gather a broad range of perspectives, as they facilitate the exchange of ideas and encourage participants to build on one another’s inputs. We anticipated that the focus group interviews would foster a rich discussion, generate diverse viewpoints, and encourage participants to contribute more than would be possible through individual data collection. The multidisciplinary composition allowed for capturing individual opinions while promoting dynamic interaction [27]. This approach proved particularly advantageous when presenting a prototype version of the app, as the group discussions were dynamic, evolved over time, and brought valuable insights. The compositions of the focus groups are summarized in Multimedia Appendix 1.

Recommendations for focus group sizes vary, with some suggesting groups of 4 and others suggesting up to 20 participants [28]. Effective groups should be large enough to generate rich data but small enough to allow active participation. Our sample size was chosen to balance the diversity of perspectives with the depth of discussion. Data collection continued until thematic saturation was reached, when recurring insights emerged consistently across groups, suggesting no new information would be gained [29]. This approach supports the credibility and trustworthiness of our findings.

Participants were recruited through a combination of methods. IT experts and health personnel were recruited through direct invitations from the research team. Patient recruitment for this study was facilitated in collaboration with the Norwegian Spine and Back Pain Organization, using their extensive network. The project was advertised on the organization’s website and at various events, and individuals expressing interest in participation were directed to contact the research team. The organization serves as a patient organization dedicated to ensuring individuals receive appropriate treatment and represents patients’ rights within the Norwegian health care system. Ultimately, 5 adolescent representatives, each accompanied by a parent, and 3 trained adult user representatives from the Norwegian Spine and Back Pain Organization were enrolled. Adolescents from diverse cultural and socioeconomic backgrounds were included in the recruitment process. Eligible participants were individuals diagnosed with AIS who had undergone either conservative treatment or surgical intervention, with 1 participant under observation only.

### Data Collection

Data were collected by conducting focus group interviews as workshops in English and Norwegian in the spring and summer of 2023. The interview and workshop guides (Multimedia Appendix 2) were generated and validated together with clinical scoliosis experts from Norway. Each workshop extended for approximately 3 hours, with a short break included. All sessions were recorded with the explicit consent of the participants. Adolescents younger than 18 years attended alongside their parents, and all participants provided written consent prior to participation. An interview guide, featuring semistructured questions, was collaboratively developed by the research team in conjunction with medical experts specializing in scoliosis treatment from Norwegian hospitals and user organizations (refer to Multimedia Appendix 2 for details). Two researchers acted as facilitators during the workshops. Participants could attend either in person or virtually in a hybrid format.

### Ethical Considerations

The study received approval from Sikt, the Norwegian Agency for Shared Services in Education and Research (reference number: 743837). Written consent was obtained from all participants prior to their involvement in the study. All steps and methods were performed in accordance with relevant guidelines and regulations. Ethical approval was deemed unnecessary for this research, according to the decision of the Regional Committees for Medical and Health Research Ethics in Norway (REK; case number: 584004).

### Data Analysis

Focus group discussions were audio recorded, transcribed verbatim, and translated into English where necessary. Data were analyzed using a structured content analysis approach [30] in NVivo R1 (2020; Lumivero) and Excel (Microsoft Corp). Initial coding was performed by the first author (KS), and a second researcher (AM) independently coded a subset of transcripts to assess interrater reliability, with discrepancies resolved through consensus. Thematic saturation guided the endpoint of the analysis. Co-authors reviewed themes to ensure consistency, enhancing the analytical rigor and credibility of the findings.

## Results

### Key Themes

**Figure 1:**
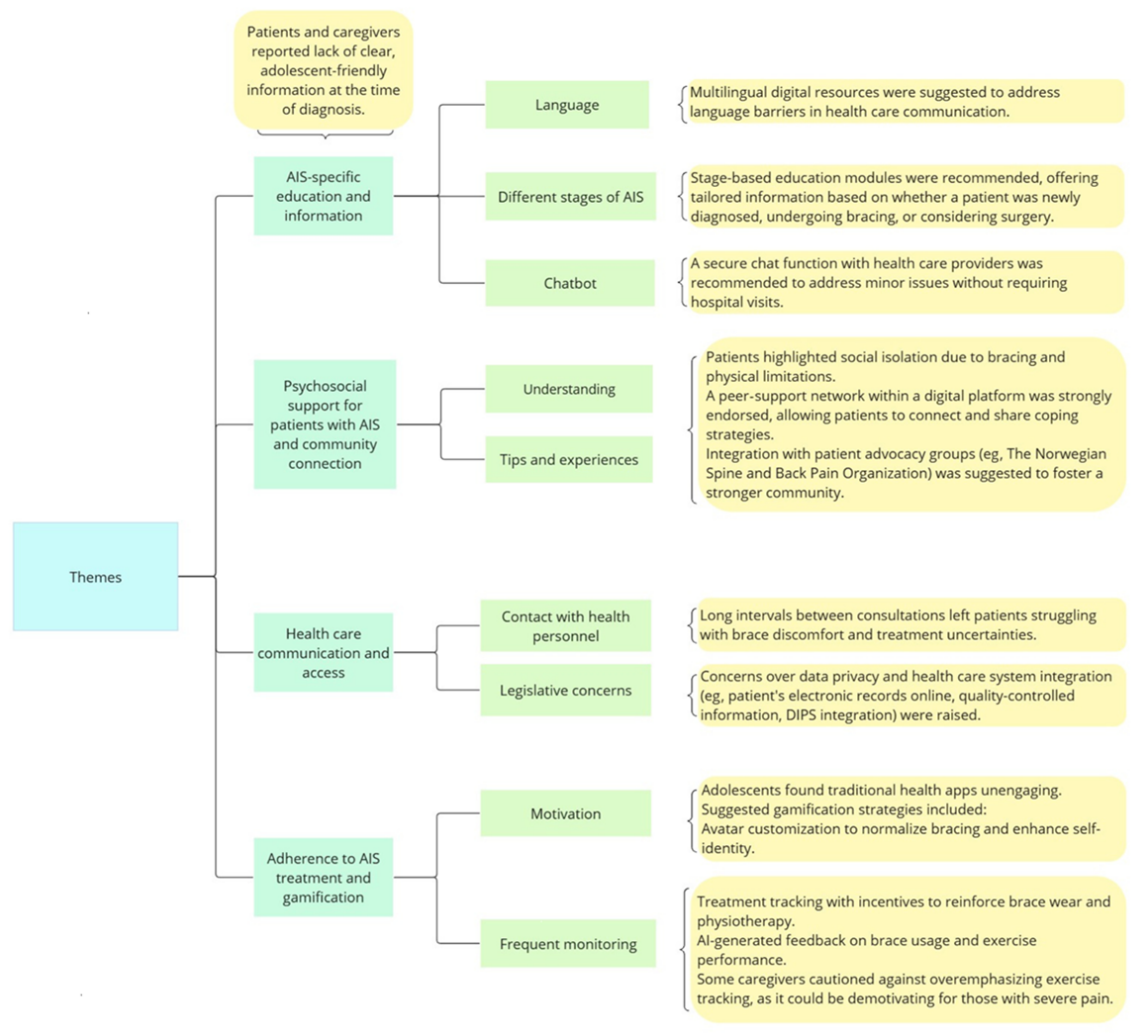
Themes and subthemes that emerged from data analysis, with short descriptions capturing the main concern of each topic. AI: artificial intelligence; AIS: adolescent idiopathic scoliosis; DIPS: Distributed Information and Patient Data System in Hospitals (Distribuert Informasjons- og Pasientdatasystem i Sykehus).

The analysis was conducted to identify the final themes and subcategories. Based on data analysis, the following themes were generated (Figure 1): (1) AIS-specific education and information, (2) psychosocial support for patients with AIS and community connection, (3) health care communication and access, and (4) adherence to AIS treatment and gamification. Some of the participants’ quotations are presented to closely describe individual themes.

Two versions of different prototypes of a potential smartphone app and related content (Multimedia Appendices 3 and 4) were created and presented to the participants in the workshops. The first prototype was discussed in the fourth focus group interview. Based on the participants’ feedback, an edited version of the prototype was presented during the fifth focus group interview, and new comments were obtained. No usability testing was performed. Overall, the feedback on the prototype was positive as it corresponds to a portable, flexible, and accessible solution.

### Theme 1: AIS-Specific Education and Information

Patients and their caregivers shared their experiences when receiving an AIS diagnosis, noting that health personnel often lacked sufficient knowledge about the disease to provide proper information. One patient shared a less-recognized situation where her general practitioner did not know anything about scoliosis, a situation rarely observed in other chronic conditions, where doctors are typically well-informed. Understanding the patient’s situation when receiving an AIS diagnosis is essential, not only for further cooperation but also because prepared patients are recognized to have better outcomes, which decreases the cost of health care services.

I think a well-prepared patient will have better outcome, will get home much earlier and in a safer way.

Physiotherapist

The aim of implementing the potential app is also to allow access to information at any time. Patients after surgery usually do not remember the given instructions properly. Parents admitted that they felt overwhelmed when receiving the diagnosis of their children and were not able to listen to the information.

#### Subtheme 1.1: Language

Health personnel acknowledged that they encounter situations where they lack a common language with patients. In such cases, effectively communicating information and preparing patients for their new life circumstances related to their diagnosis becomes nearly impossible. Therefore, they were very pleased with the solution of providing information in different languages, with Urdu, Arabic, and Polish being the most frequent.

But you also maybe need to do something in a lot of languages then… And then you can have Urdu, you can have Arabic. Yeah, you can have… You have a lot of Polish, earlier Russian or the Balkan languages.

Physiotherapist

#### Subtheme 1.2: Different Stages of AIS

Patients seek help throughout their journey, and information should be tailored to their current needs.

I think that's important when you can… you can kind of build those different phases or that... that they would, would… program also would change in... in what kind of situation you are in and you can have some when you have the information exactly for your situation.

IT expert

As AIS represents diverse conditions for each patient, the app should offer different information for various stages of a patient’s journey: just received a diagnosis, undergoing surgery, following brace treatment, etc.

Well, really, I think… that if it could be divided up… whether you need softening, pain relieving exercises for… in the shoulders, or the pelvis, back… Yes, it could be divided a bit according to different types of needs. It’s a bit as if, whether it is training or whether it is just painful muscles and softening exercises… Yes, like specific exercises or, yes, for the program, some exercises for the program.

Family caregiver

#### Subtheme 1.3: Chatbot

Patients represented the adolescent generation that is well adapted to digital technology use. Therefore, when discussing the form of providing information, a chatbot was suggested and well-received by patients, as the young generation is very familiar with this tool. An IT expert and health personnel admitted that talking to health personnel may be difficult, as it would require a report in the patient’s health care journal, and there are other legislative obstacles regarding implementation.

Well, I think in a way to get an answer to what you're wondering about. There can be so many different... Yes, from where it hurts or what... Yes, one or the other. That one can search up own questions. If it’s possible to add some information? Some concrete information that I can search up. For example, in Helsenorge I can ask questions my GP. Is that possible?

Patient

Yes, it is. Absolutely.

Researcher

Yes. Otherwise, I can't think of anything else right now... Maybe tips about activities that can be okay, or... Or training, possibilities, or...

Patient

Once we started using chatbots, they were very happy because… They’re quite used to talking to chatbot, right? They’re very used to that, so this is a very known way of interacting.

Researcher

This is a very good suggestion. That you can... That activities can, in a way, become tips from other users. Where you write... So, for example, as XXX has suggested, swimming and stretching is something that maybe not everyone think of...

Another patient

### Theme 2: Psychosocial Support for Patients With AIS and Community Connection

The biggest theme that emerged from the group discussion was the need for support. This could be facilitated by a smartphone app dedicated to AIS, where patients could connect and create their own community. According to reports from our research participants, they would have appreciated communication with others experiencing scoliosis, especially their peers. It would be valuable for them to listen to patients after surgery or those undergoing brace treatment for several years to understand the severity of their own situations and eventually increase treatment compliance, which is important for AIS. Patients have questions they may be too shy to ask at the doctor’s office.

So what I missed. was someone to be with. Who had gone through the same thing. It did not have to be specific. But someone who could understand me to a certain extent.

Patient

#### Subtheme 2.1: Understanding

Receiving an AIS diagnosis may result in the patient experiencing detachment and isolation from their existing social network. Patients often report feelings of alienation, expressing a lack of available confidants. Through the app, connecting with fellow patients offers a greater opportunity for empathy, understanding, and exchange of emotions and perspectives. Participants expressed their wish to have this app connected with “Ryggforeningen i Norge” (the Norwegian Spine and Back Pain Organization for scoliosis), which has a very active approach. The association organizes events that allow patients to meet, connect, and create their own community. The discussion provided participants with the opportunity to express their preferences for the digital solution.

Connect the app with the patient association.

Family caregiver

I got that understanding when I went to xxx and met others who had experienced the same as me. A group of people with scoliosis in different ways.

Patient

#### Subtheme 2.2: Tips and Experiences

According to the patients and their parents, it would be beneficial to be provided with tips and experiences specific to AIS from patients who have been or are in the same situation. Participants mentioned areas like school (eg, having 2 sets of books to avoid carrying heavy backpacks, getting extra time for exams, and the right to be driven to school by taxi) and types of aids that do not come to mind.

Like for example, two sets of books and not have to carry a heavy bag to and from school, and others, like chairs to sit on, and yes, and stuff like that.

Family caregiver

They would also appreciate recommendations for physiotherapists experienced with AIS or tips for general physical activity suitable for AIS, as some participants admitted that not all physiotherapists are familiar with PSSEs. They mentioned sharing exercises and workout tips that are safe for a patient’s back.

And I also think that what you mentioned about yoga, I think many have benefited from it. I have not done it myself, but I have heard that it is nice if you have scoliosis.

Patient

Some patients revealed their daily struggles after surgery. For example, they could not comfortably lie down or sit. Thus, advice in this direction would be greatly appreciated. During the group discussion, several ideas were revealed. Some questions included “What type of chair or mattress do they use?” and “What tips do they have for lowering the pain?”

When I got surgery, I found very useful tips for such a little thing, for example how to lay comfortably in a bed again. One has to find out how to sit at the dinner table. Just the practical tips one may think are not needed.

Patient

### Theme 3: Health Care Communication and Access

The implementation of the app aims to lower the worldwide burden on the overloaded health care system and facilitate smoother connections between patients and health personnel. This is expected to reduce the frequency of personal visits to the doctor.

You can take the app with you into the consultation room and share it should you want to. And I think that's kind of important also is… That it clearly communicates this is a patient’s choice.

IT Expert

#### Subtheme 3.1: Contact With Health Personnel

Especially with AIS, the period between checkups is sometimes 6 months. During this period, patients can experience discomfort when wearing a brace and need detailed guidance from health personnel. Long waiting times to resolve a patient’s problem lower the effectiveness of treatment. According to the participants, it would be beneficial to have the opportunity to ask their doctors or physiotherapists about a current problem so they could continue to follow up on the treatment plan. Patients sometimes have simple questions that could be easily communicated through an app.

If I could have a wish, it would be in relation to the interaction with the health care system.

Family caregiver

Patients and their caregivers also wished to receive information digitally and not on a piece of paper. The app could serve as a storage tool for their rehabilitation exercises, which would be easily accessible at any time.

#### Subtheme 3.2: Legislative Concerns

There are legislative concerns requiring further examination, particularly regarding the conversation between patients and medical staff, which must always be documented in the patient’s journal. As a result, some of the proposed ideas were immediately dismissed. An IT expert who was familiar with legislative concerns attended the focus group interviews.

They said no completely to everything because you cannot have an app that’s owned by the hospitals where you don’t have 100% control everything.

IT expert

A similar obstacle emerged when providing quality information to patients through an app, which would require control of all shared content by health personnel. For now, this appears too time-consuming, as acknowledged by 1 health care professional:

Very important for… for good and safe treatment is that we all give answers to questions, so it will be kind of heavy work for the health personnel to follow this.

Physiotherapist

Another concern involves integrating the Distributed Information and Patient Data System in Hospitals (DIPS; Distribuert Informasjons- og Pasientdatasystem i Sykehus), a robust, reliable electronic patient record system used in Norwegian hospitals, into the app.

You know anything that doesn’t match with the DIPS doesn’t match. DIPS isn’t a… DIPS is a big problem. So, if this should be used by us, it must be on the patient’s own platform as the tools to as you help yourself.

Physiotherapist

### Theme 4: Adherence to AIS Treatment and Gamification

An AIS diagnosis often marks the start of a long-term condition, making it challenging for patients to follow a treatment plan. Bracing requires several years, and physiotherapy is sometimes a lifetime regimen.

This is so important to have a toolset like yours to be able to keep the communications up with the patient for a long time and… It‘s scoliosis. I see that it's not months, it's years. It's maybe for the rest of their lives.

IT expert

#### Subtheme 4.1: Motivation

Introducing an app to patients is expected to increase treatment compliance and enhance their motivation through gamification. The interactive discussion proved to be very convenient, as the patient sample in our research was exclusively female (the prevalence of AIS is significantly higher in females), and helped us to understand that the suggested games were not interesting features for our participants.

Researcher: Do you have any games you play?

Patient 1: No.

Patient 2: No, not at all.

The focus groups revealed that traditional game-based gamification was not appealing to adolescent girls with AIS. This feedback prevented the development of a misaligned app based on incorrect assumptions. Patients rejected a feature (games) but showed interest in another (avatars), and this demonstrates that user needs should guide app development. The discussion directly inspired an alternative gamified feature that better aligns with their preferences. A physiotherapist’s comment suggested that avatars could be a positive and engaging feature for patients, especially since they enjoy customizing aspects of their treatment, such as brace design. This aligns with the idea that different forms of gamification, like customization and point-based rewards, might be more effective than generic gaming elements.

I think the avatar sounds pretty… I… I think my patient would like that, because they are very into designing like how the braces look like, the pattern.

Physiotherapist

Gamification can be approached by receiving points from workouts or alternative activities and saving the results in the app. By sharing activities, patients can get inspired and develop an interest in different activities they might not think of as safe choices (eg, yoga, swimming, skiing, and meditation). These must be carefully considered in terms of contraindications and the medical condition of each patient. If the app allows tracking progression and provides feedback to patients, it could also serve as a motivating feature.

Caregivers’ concerns about exercise-focused gamification potentially demotivating some patients further reinforce the need for an adaptive design approach. This highlights the importance of user-centered design for patients with AIS and iterative testing through discussions with the target group.

So, if it’s: Now you have managed this much when you exercise, I understand because then you can manage it. But it can be demotivating, and it can be counterproductive when the person can’t manage to do it.

Family caregiver

#### Subtheme 4.2: Frequent Monitoring

The app is meant to allow patients to monitor their situation over a longer period. Participants suggested including a diary feature, which would be available to store information regarding their pain, sleep, treatment, mood, feelings, or any other aspect. This results in an overview that makes it easier to detect any changes in pain, problems, etc. The diversity of our interview group helped us gain a better understanding of what features would be most beneficial.

But what I think emotionally and psychologically about this diary is that I believe that if they have a place to put all their thoughts and feelings and write it down, we have that… And I think if we… if we have a system that organized in that way that you can put all the, that's that one place. That could be therapy in itself.

Physiotherapist

This is expected to not only increase treatment adherence but also provide more accurate information about the patient’s history. Recall of events appears to be uncertain. This feature may be helpful regarding the balance between pain and exercise.

So there are also a lot of things I don't remember from the hospital stay. Because of my pain meds...

Patient

So, so in that stage, when they are lying there in the hospital and they are waking up, they... they would need this kind of basic information like what, what can I do now and... and how to how to... And... and they... they lie in bed and they have this devices, they can go back or what did she say? I don't remember. And maybe my parents are coming here. Have the doctor been here? Have had the physio being here? Yes, but I don't remember a thing.

Physiotherapist

## Discussion

### Principal Findings

This study explored patients’ and caregivers’ perspectives on a potential digital solution for managing AIS and identified 4 key themes based on participants’ perspectives. The themes of *AIS-specific education and information*, *psychosocial support for patients with AIS and community connection*, *health care communication and access*, and *adherence to AIS treatment and gamification*, emerging from the conducted interviews, highlight how such an app could significantly enhance the treatment process for patients. These themes reflect common concerns in chronic disease management, such as the need for accessible information, peer support, and tools that enhance treatment adherence. The results of the study support findings from other studies on the potential for digital health technologies to improve conservative treatment for AIS, specifically through a smartphone app [21,31-33]. However, an appropriate way to educate children, adolescents, and young adults about age-related problems due to scoliosis has not yet been found [34].

Several studies have been conducted related to technologies that can improve AIS treatment, such as the one by Bottino et al [21], which evaluated 6 apps for scoliosis management. The presented apps were mostly designed to measure, record, and track AIS periodically. In our approach, we did not prioritize the measurement feature, but rather our focus was on simplifying patients’ AIS journeys and enabling them to track their treatment progress. Several of the apps highlighted in the study by Bottino et al [21] enable tracking. The APECS app stores exercises prescribed by doctors, and ScolioTrack provides answers to frequently asked questions about scoliosis, featuring functionalities we aim to incorporate into our solution. We identified some similarities with the study by Bottino et al [21], as the use of the app should bring advantages to both patients (self-care management strengthens patients’ involvement in treatment) and health professionals (reducing workload as it is easier to track appointments in the app and allowing remote communication with patients). The Scoliosis Tracker app includes educational content and appointment reminders, which align with the key features anticipated by our participants. Based on our results, there is a need to provide psychological and social support features that are not present in any of the introduced apps [21]. Similarly, other studies have described the wish of patients with chronic diseases to use the app to increase treatment adherence [35].

### Education and Information

Our study has shown the challenges patients with AIS and their caregivers face in receiving adequate information, particularly at the time of diagnosis. Our findings are in line with the results of a systematic review by Motyer et al [36], where more than 70% of parents of patients with AIS lacked information on topics such as curve progression. One participant in our study reported how her general practitioner was unfamiliar with scoliosis, leaving the family without proper guidance, which highlights the essential need for support among patients with AIS. This can cause a critical gap in patient education, which can undermine the entire treatment journey. Understanding the disease itself is essential for successful further treatment. Patients who are well-informed tend to have better health outcomes and require fewer health care services, leading to reduced costs. Previous research has similarly highlighted that patient education significantly influences treatment outcomes and quality of life [37]. Cho et al [19] analyzed the functionality of existing scoliosis apps that improve brace-wearing compliance, where only 5 out of 10 apps provided educational information to users, and 3 out of 10 apps provided general information on scoliosis. In our study, we propose an app that would allow patients to access AIS information at any time, as it has been conveyed that patients and their caregivers are often overwhelmed when receiving the diagnosis and are unable to take in more information. Our results correlate with the findings of the study by Cho et al [19], as health personnel also shared the need to have the app accessible in several languages, since there is sometimes no common language between them and their patients, resulting in a lack of information being provided by health personnel. The study by Bottino et al [21] similarly underscores the importance of web-based applications, such as Scoliosis Manager, which is accessible in 10 languages. In alignment with these findings, our study further identifies multilingual accessibility as a key feature desired by participants. The proposed app would address this by offering reliable, accurate information available in multiple languages (such as Urdu, Arabic, and Polish), ensuring that patients from diverse backgrounds can access essential information. Cho et al [19] highlighted the significance of both language accessibility and the necessity for the app to present information in a manner that is comprehensible to individuals with limited health literacy. As patients with AIS represent the young generation, a chatbot was suggested as an adequate form of providing information through the app. This feature could be particularly effective for addressing specific challenges faced by patients with AIS and providing them with support during the long waiting time between doctor’s appointments, offering real-time feedback and immediate answers to their questions comprehensively [38].

### Artificial Intelligence and Personalization

In the field of AIS, the use of large language models (LLMs), like advanced artificial intelligence (AI)-driven chatbots, is transforming patient education, engagement, and the way information is shared [38,39]. Given the complexity of AIS and its diverse treatment options, LLM-driven chatbots can deliver personalized, medically accurate, and easily comprehensible information, enhancing patient autonomy and informed decision-making. Furthermore, LLMs can adapt responses based on the user’s knowledge level and emotional state, ensuring a more supportive and empathetic interaction. This is particularly valuable in AIS management, where patient comprehension and engagement are critical for successful treatment adherence. By alleviating anxiety and promoting a participatory health care model, the use of LLMs in education for patients with AIS could lead to higher patient satisfaction and improved treatment adherence, thereby contributing to better long-term health outcomes [39]. However, these systems need to be rigorously tested and validated before implementation to ensure their accuracy, effectiveness, and ethical compliance in real-world health care settings.

Additionally, the discussion revealed that patients, caregivers, and health personnel all agree that the app should be tailored to individual needs and the patient’s current stage of AIS treatment, which correlates with findings reported in a study by Schwieger et al [40]. By providing stage-specific content, whether for newly diagnosed patients, those undergoing surgery, or those following conservative treatment, the app should ensure that the information remains relevant and actionable throughout the patient’s journey. The importance of language and personalized information aligns with research suggesting that tailored patient education can improve treatment adherence [41]. Unlike other chronic conditions, AIS demands a high level of adherence to rigid bracing and long-term physiotherapy, making customization essential for patient engagement and compliance.

While several smartphone apps exist for scoliosis management, they primarily focus on basic tracking and assessment, such as measuring spinal curvature or monitoring treatment adherence. However, there is a notable lack of personalized, interactive solutions tailored to individual patient needs. Current apps, such as ScolioTrack and Scoliometer, provide monitoring functionalities but do not offer adaptive, patient-centered treatment guidance [21]. To our knowledge, no existing scoliosis app integrates personalization, gamification, and AI-driven decision support to enhance patient engagement and adherence. This gap highlights the novelty of our approach and underscores its potential impact on scoliosis treatment.

### Community Support

Beyond education, the study also underscores the emotional isolation many patients feel after receiving an AIS diagnosis. Unlike individuals with chronic conditions, patients with AIS lack natural peer groups for support [42]. This sense of detachment from their social circles can worsen the psychological toll of the condition. Participants in our study expressed a strong desire for peer-to-peer support, where patients could connect with others facing similar challenges. Our results align with the findings of Wellburn et al [42], suggesting the need for support resources centered on facilitating contact with individuals who have undergone similar experiences of AIS, as well as sharing success stories.

The app seems to be an appropriate way of communication for typically young patients with AIS and could provide a vital community-building platform, offering a space for patients to share experiences, offer practical advice, and support one another through difficult phases of treatment [40,42]. For instance, patients suggested linking the app with patient associations, which already foster scoliosis communities and organize events, and are positively received among patients with AIS. The connection with such organizations further highlights the value of structured peer support systems in AIS management, aligning with the findings reported by MacCulloch et al [43]. However, there is currently no existing research specifically focused on using digital technology to establish or support peer-to-peer connections.

This could enhance the sense of belonging and support that many participants identified as essential during the discussion. Sharing personal tips, specific to patients with AIS, such as strategies for managing pain after surgery, adequate physical activity or sports, or strategies in school settings (eg, extra time for exams or 2 sets of textbooks to avoid carrying heavy bags), could also alleviate some of the day-to-day struggles patients and their caregivers face, promoting better treatment compliance, which is essential for good treatment outcomes in AIS management. The need for community support indeed echoes findings from chronic disease literature, where peer interaction has been shown to reduce feelings of isolation and improve psychological well-being [36,43]. Our findings support the potential for an app that would foster AIS community interaction, allowing patients to share experiences and advice, which can empower them in managing their condition [40]. However, the digital community platform should be tailored specifically to the needs of patients with AIS to improve their mental well-being [42].

Building community support for a gamified scoliosis app presents unique challenges, particularly regarding regulatory compliance, privacy, long-term engagement, and misinformation management [40,44,45]. Prior research and interviews with stakeholders indicate that regulations can restrict the level of peer-to-peer interaction, requiring careful design choices to balance social engagement with data protection laws [46-48]. Additionally, to mitigate the risks of misinformation and disengagement, the app could incorporate verified educational content, professional moderation, and adaptable gamification settings [19,31,49]. Future work should explore user feedback mechanisms and co-design with patients and clinicians to refine the community-building aspects while ensuring compliance and ethical responsibility.

### Health Care Services

Another crucial aspect is how the app could ease the strain on health care services [21]. Patients with AIS often go months between check-ups, and during this time, they may experience discomfort or require guidance on wearing braces or performing exercises, as shared by our focus group. This app could allow patients to contact health care professionals for quick answers to minor issues regarding their current treatment method, reducing the need for unnecessary in-person visits. Research by Schwieger et al [40] explored the most sought information online by adolescents with AIS and their parents, which included causes, progression, diagnosis, brace treatment of AIS, and others’ experiences. Participants’ desire for improved communication with health care professionals is a recurring theme in the health care literature. The long intervals between appointments for patients with AIS often leave them seeking more frequent guidance, which has been noted in other chronic conditions such as diabetes [50,51]. Detailed features of the app should be discussed in the future to meet the individual medical needs of patients, their cultural and social background, their caregivers, and health care professionals.

The app also offers a modern communication tool, replacing outdated paper flyers [52]. However, there are legislative concerns regarding documentation, integration with electronic health records (such as the Norwegian DIPS system), General Data Protection Regulation (GDPR), ethics, fairness, and the general safety of using digital applications in the health care practice [26]. Therefore, not all the features proposed in the discussion could be integrated into the actual app, as was learnt from the IT specialist. These challenges should be resolved to ensure secure and legally compliant interactions between patients and health care providers, as all the information provided by health care professionals needs to be documented in patients’ journals. It was confirmed by one of the health personnel that updating relevant information in patients’ medical records is excessively time-consuming. Nonetheless, streamlining patient-provider communication through the app could likely enhance the effectiveness of the treatment plan and reduce patient anxiety during long periods between appointments.

### Treatment Compliance and Motivation

In addition to health care services, the importance of treatment compliance and motivation emerged strongly from participant feedback. The issue of treatment compliance is central and specific to the management of AIS, as long-term adherence to bracing and physiotherapy is often required. To address this, the app should incorporate gamification features to encourage compliance. Findings from our study align with the growing body of research on the role of digital tools in enhancing treatment compliance [53,54]. Group discussion introduced diverse ideas, as some participants expressed disinterest in gaming, echoing similar findings to those of Günther et al [34], who described that the greatest uncertainty in combining exercises with a game was likely due to the lack of a digital reference and participants’ difficulty imagining their therapy in a gamified format. The study by Günther et al [34] suggested that playful elements can aim to boost motivation through game enjoyment and digital rewards (eg, badges and points). In our study, participants were intrigued by features like creating avatars and other gamification elements, such as earning points for completing exercises or engaging in activities like yoga or swimming. Högberg et al [55] identified a framework consisting of 6 gamification dimensions: accomplishment, challenge, competition, guidance, playfulness, and social experience, which further expands upon the results of our findings. As reported by Jansson et al [56], from health care professionals’ perspectives, the greatest gamification opportunities lie in personalized counseling, monitoring, and social support. These were also emphasized as desired features by our participants and should be implemented in the final solution. Additionally, various game elements were found by Jansson et al [56] to support these experiences (eg, accomplishment, activity tracking, awarding points, reminders, visualizations, etc). Some of the findings correlate with the results of our research. However, that study revealed a broader range of new opportunities that were not identified in our study, for example, increasing the difficulty of challenges, competition, and comparison, which should be carefully implemented, as there could be both negative and positive effects.

It is essential to ensure that all exercises provided via the app adhere to the contraindications specific to each patient’s medical condition [34]. Cho et al [19] presented examples of scoliosis apps in their study that incorporate PSSEs and provide feedback. The use of avatar customization in apps specific to AIS can address body image concerns and help patients accept bracing as part of their identity [57]. In the current literature, features, such as avatars and point-based systems, have been shown to increase patient engagement in other health-related apps. Furthermore, the acceptance of therapeutic support may be increased through the incorporation of gamification elements (eg, quizzes), as suggested by the research of Dannehl et al [52]. Wibmer et al [11] specifically explored the potential of gamification in scoliosis therapy, demonstrating its ability to enhance both motivation and precision in performing scoliosis-specific exercises.

Additionally, the app could include a diary feature to help patients track their pain, sleep, and overall progress, fostering consistent adherence related to the AIS treatment plan over a long period. However, one of the interviewed participants pointed out that the need for balance is critical to ensure the app remains motivating. Overly focusing the app on exercise tracking could potentially discourage patients with physical limitations (eg, in the postoperative period), a concern noted by caregivers during the interviews. The diary can provide easier detection and explanation of any changes (pain, sleep, treatment, etc). This is anticipated to not only enhance treatment adherence unique to AIS but also yield more accurate information about the patient’s history, as the recall of events seems to be uncertain [58]. This feature may prove beneficial in balancing pain and exercise.

A prototype of the app (Multimedia Appendix 3) was presented to the participants in our study. Based on constructed discussions in our group, the following gamification elements seem to be valuable for patients with AIS: an avatar, a diary, a chatbot, a feature providing information and exercises related to AIS, and an element related to peer support [40]. The interviews revealed that this digital platform should also provide tips relevant to the daily lives of patients with AIS. In further steps of designing and developing a solution, these elements can differ.

### Implications for Theory

The findings of this study have several theoretical implications that extend beyond the immediate context of AIS management. First, the study contributes to a broader understanding of how digital health technologies can be effectively integrated into chronic disease management. By identifying the specific needs and preferences of patients with AIS and their caregivers, the study provides information that can be adapted to other chronic conditions [32,59].

Moreover, the study revealed the critical role of cultural and linguistic inclusivity in health technology design. The inclusion of multilingual support in the app underscores the necessity of developing health technologies that are accessible to diverse patient populations. This finding aligns with the growing body of literature advocating for culturally competent health care practices and suggests that future research should continue to explore how digital health tools can be designed to meet the needs of diverse user groups [60].

The study also advances the theoretical understanding of gamification in health behavior change. By demonstrating the potential of gamification elements to enhance treatment adherence among patients with AIS, the study provides empirical support for the use of game-based strategies in health interventions. This insight can support future theoretical models of health behavior change, particularly in the context of chronic disease management, where long-term adherence to treatment plans is crucial [61,62].

### Implications for Practice

The integration of digital health technologies into AIS treatment presents a promising avenue for improving patient education, community support, health care services, and treatment compliance. The findings of this study underscore the importance of addressing the specific needs of patients with AIS and their caregivers through tailored, accessible, and culturally appropriate digital solutions. While significant challenges remain, particularly regarding legislative and logistical barriers, the potential benefits of such technologies are substantial.

Practically, the study offers several actionable insights for health care providers, technology developers, and policy makers. For health care providers, the study underscores the importance of comprehensive patient education and the need for continuous support throughout the treatment journey. The development of a digital app that provides reliable, accessible information can help bridge the knowledge gap identified in the study, ensuring that patients and their caregivers are well-informed and better prepared to manage AIS [25].

For technology developers, the study provides a clear roadmap for designing effective health apps. The emphasis on patient-centered design, multilingual support, and gamification features offers practical guidelines for creating digital health tools that are both engaging and effective. Developers should prioritize these elements to enhance user engagement and treatment adherence, ultimately improving health outcomes [63,64].

Policy makers can also draw valuable lessons from this study. The legislative and logistical challenges highlighted in the research point to the need for clear regulations and streamlined processes to facilitate the integration of digital health technologies into existing health care systems. Policy makers should work toward creating an enabling environment that supports the adoption of digital health tools, ensuring that they are secure, legally compliant, and seamlessly integrated with electronic health records [65].

Additionally, the study’s findings on the importance of community support suggest that health care systems should incorporate peer support mechanisms into their service delivery models. By fostering connections between patients through digital platforms, health care providers can enhance the psychological well-being of patients and improve treatment adherence. This approach can be particularly beneficial in managing chronic conditions, where long-term support and motivation are essential [66].

In conclusion, this study provides valuable theoretical and practical insights into the integration of digital health technologies in AIS management. By addressing the specific needs of patients with AIS and their caregivers, the study offers a comprehensive framework that can inform future research and practice in the field of digital health. The implications for theory and practice underscore the potential of digital health tools to transform chronic disease management, improving patient outcomes and enhancing the overall quality of care.

### Limitations

Several limitations of this study should be acknowledged. First, the data collection relied on focus group interviews, which may have introduced bias due to group dynamics. Participants might have felt pressured to conform to dominant opinions rather than express their true experiences. Individual interviews could have provided deeper insights by allowing for more personal reflections. Second, the study was conducted using a small sample size, limiting the generalizability of the findings. While the focus groups provided valuable insights into the perspectives of patients with AIS and caregivers, a larger and more diverse participant pool could yield more comprehensive results. Third, the recruitment process primarily involved a single organization, the Norwegian Spine and Back Pain Association, which provided access to a specific network of participants. While this facilitated recruitment, it may have influenced the perspectives shared during focus group interviews. All participants were female, which may limit the generalizability of the findings in contexts where gender differences are relevant. However, this sample composition reflects the higher prevalence and treatment rates of AIS among females [3]. Additionally, recruitment through personal invitations may have introduced selection bias, as individuals with greater motivation or prior interest in the topic may have been more likely to participate. These factors could have affected the diversity of the opinions and experiences captured.

Fourth, the digital solution was only presented conceptually, meaning that feedback from participants was based on a conceptual understanding rather than actual long-term use. Without feasibility studies and real-world testing, it is difficult to determine the app’s practical effectiveness or its ability to meet the identified needs.

Fifth, the study focused primarily on patients and caregivers within the Norwegian health care system, which may not reflect experiences in other health care settings. Consequently, the findings may not be generalizable to a global population. For instance, views on access to information or health care support may vary depending on available resources in different countries. Additionally, AIS requires individualized treatment approaches, meaning that the content of digital health solutions must be adapted to each patient’s specific needs [42,67]. Furthermore, while legislative and regulatory concerns regarding health care apps were briefly mentioned, this study did not explore them in depth. Future research should examine legislative barriers to integrating digital tools into health care systems. Finally, usability testing was not conducted. Subsequent studies could benefit from broader recruitment strategies involving multiple organizations or varied methods of recruitment to ensure a more diverse sample. Future research should focus on developing and testing digital solutions in real-world settings to evaluate their effectiveness and feasibility. Addressing the identified barriers will be crucial to ensuring the seamless integration of digital health technologies into existing health care systems. This approach can enhance the quality of care for patients with AIS and better support them in managing their condition.

These identified challenges align with our research agenda, where our group is actively working on exploring legislative barriers, conducting usability testing, and developing evidence-based digital solutions to enhance the care of patients with AIS and their integration into health care systems.

### Conclusion

The findings of our research make a significant contribution to the field by highlighting the challenges faced by patients with AIS and their caregivers, offering new perspectives of understanding in terms of access to education, community support, health care services, and treatment compliance. The findings emphasize the need for improved communication and information-sharing platforms that can offer timely, personalized guidance to patients at various stages of their treatment journey. A smartphone app designed to address these needs was positively received by participants, who valued the potential for better access to information, peer support, and closer interaction with health care professionals.

The identified themes suggest that digital tools, if properly implemented, can play a critical role in alleviating the emotional burden associated with AIS and enhancing treatment compliance through personalized support and motivational features. However, the study also highlights potential legislative and logistical barriers that need to be addressed before such solutions can be fully integrated into health care systems.

The results indicated that the development of the digital solution holds significant potential to enhance the conservative treatment of AIS. By addressing key issues, such as education, community support, and health care communication, and by encouraging compliance through innovative features, the app could improve patient outcomes while reducing health care costs. However, it is vital that the app continues to evolve based on users’ feedback to ensure it remains relevant, effective, and inclusive. The addition of a chatbot, as suggested by participants, could further enhance communication, particularly for younger users accustomed to digital interfaces, making the app a comprehensive tool for managing AIS in the modern health care landscape.

While this study provides valuable insights into the needs of patients with AIS, further research is needed to address the ethical and legal concerns raised regarding app-based health care solutions. Additionally, future studies could explore the effectiveness of such digital tools in improving patient outcomes, especially in long-term treatment adherence.

Future research should focus on testing the effectiveness of digital health solutions in real-world settings and exploring how they could be tailored to meet the diverse needs of patients with AIS. Additionally, further investigation is needed to address legislative and regulatory challenges associated with integrating digital tools into health care systems. By addressing these gaps, health care providers may be able to offer more comprehensive, patient-centered care that improves both the physical and emotional outcomes of individuals living with AIS. Further studies and a user-centered design will be essential to realize the full potential of digital tools in AIS care.

## Appendix

Multimedia Appendix 1 Focus group interviews and participants.

Multimedia Appendix 2 Focus group interview guide for participants.

Multimedia Appendix 3 First version of the prototype presented to participants at workshop number 4.

Multimedia Appendix 4 Version of the prototype presented at workshop number 5. The prototype was modified based on feedback received in the previous workshop.

## Abbreviations

AI: artificial intelligence
AIS: adolescent idiopathic scoliosis
DIPS: Distributed Information and Patient Data System in Hospitals (Distribuert Informasjons- og Pasientdatasystem i Sykehus)
LLM: large language model
PSSE: physiotherapeutic scoliosis-specific exercise

## Data Availability

Data can be made available upon request.
