# Supplementary material for: Exploring Stakeholders’ Perceptions of Using Digital Health Technologies to Improve the Conservative Treatment of Adolescent Idiopathic Scoliosis: Qualitative Study
Source: J Med Internet Res. 2025 Jun 25;27:e69089. doi: 10.2196/69089 (PMC12242061; doi:10.2196/69089)
Supplement: Multimedia Appendix 1 [file jmir_v27i1e69089_app1.docx]

**Multimedia Appendix 1.** Focus group interviews and participants.

**Focus Group 1: Needs and Pain Points in Scoliosis Journey, 18-04-2023:**

Patient representatives:

| Ref no |  | Gender | Time from scoliosis treatment |
| --- | --- | --- | --- |
| 1 | Patient 1 | Female | 10-20 years |
| 2 | Patient 2 | Female | 5-10 years |
| 3 | Patient 3 | Female | 5-10 years |

Experts & Company representatives

| Ref no | Title | Role |
| --- | --- | --- |
| 4 | Physiotherapist | Working with scoliosis patients currently in Norwegian hospital 1 |
| 5 | Physiotherapist | Leading physiotherapist, responsible for scoliosis treatment in Norwegian hospital 2 |

**Focus group 2: Needs & Current Practice, Physiotherapy Focus, 25-04-2023**

Experts & Company representatives

| Ref no | Title | Role |
| --- | --- | --- |
| 4 | Physiotherapist | Working with scoliosis patients currently in Norwegian hospital 1 |
| 5 | Physiotherapist | Leading physiotherapist, responsible for scoliosis treatment in Norwegian hospital 2 |

**Focus group 3: Needs & Current Practice – Patient Perspective, 09-05-2023**

Patient representatives & family care givers:

| Ref no |  | Gender | Time from scoliosis treatment |
| --- | --- | --- | --- |
| 6 | Patient 4 | Female | 3-5 years |
| 7 | Patient 5 | Female | Ongoing |
| 8 | Patient 6 | Female | Ongoing |
| 9 | Family caregiver 1 | Female | 3-5 years |
| 10 | Family caregiver 2 | Female | Ongoing |
| 11 | Family care giver 3 | Female | Ongoing |

Experts & Company representatives

| Ref no | Title | Role |
| --- | --- | --- |
| 4 | Physiotherapist | Working with scoliosis patients currently in Norwegian hospital 1 |
| 5 | Physiotherapist | Leading physiotherapist, responsible for scoliosis treatment in Norwegian hospital 2 |
| 12 | Medical doctor | Currently giving scoliosis treatment for patients in Norwegian hospital 2 |

**Focus group 4: Vision workshop, focusing on the solution content, 06-06-2023**

Experts & Company representatives

| Ref no | Title | Role |
| --- | --- | --- |
| 4 | Physiotherapist | Working with scoliosis patients currently in Norwegian hospital 1 |
| 5 | Physiotherapist | Leading physiotherapist, responsible for scoliosis treatment in Norwegian hospital 2 |

**Focus group 5: Future Scoliosis Solution, Patient Perspective 29-08-2023**

| Ref no |  | Gender | Time from scoliosis treatment |
| --- | --- | --- | --- |
| 6 | Patient 4 | Female | 3-5 years |
| 7 | Patient 5 | Female | Ongoing |
| 13 | Patient 7 | Female | Ongoing |
| 9 | Family caregiver 1 | Female | 3-5 years |
| 10 | Family caregiver 2 | Female | Ongoing |
| 14 | Family care giver 4 | Female | Ongoing |
