# Supplementary material for: Exploring Stakeholders’ Perceptions of Using Digital Health Technologies to Improve the Conservative Treatment of Adolescent Idiopathic Scoliosis: Qualitative Study
Source: J Med Internet Res. 2025 Jun 25;27:e69089. doi: 10.2196/69089 (PMC12242061; doi:10.2196/69089)

**Multimedia Appendix 3.** First version of the prototype presented to participants at workshop number 4.


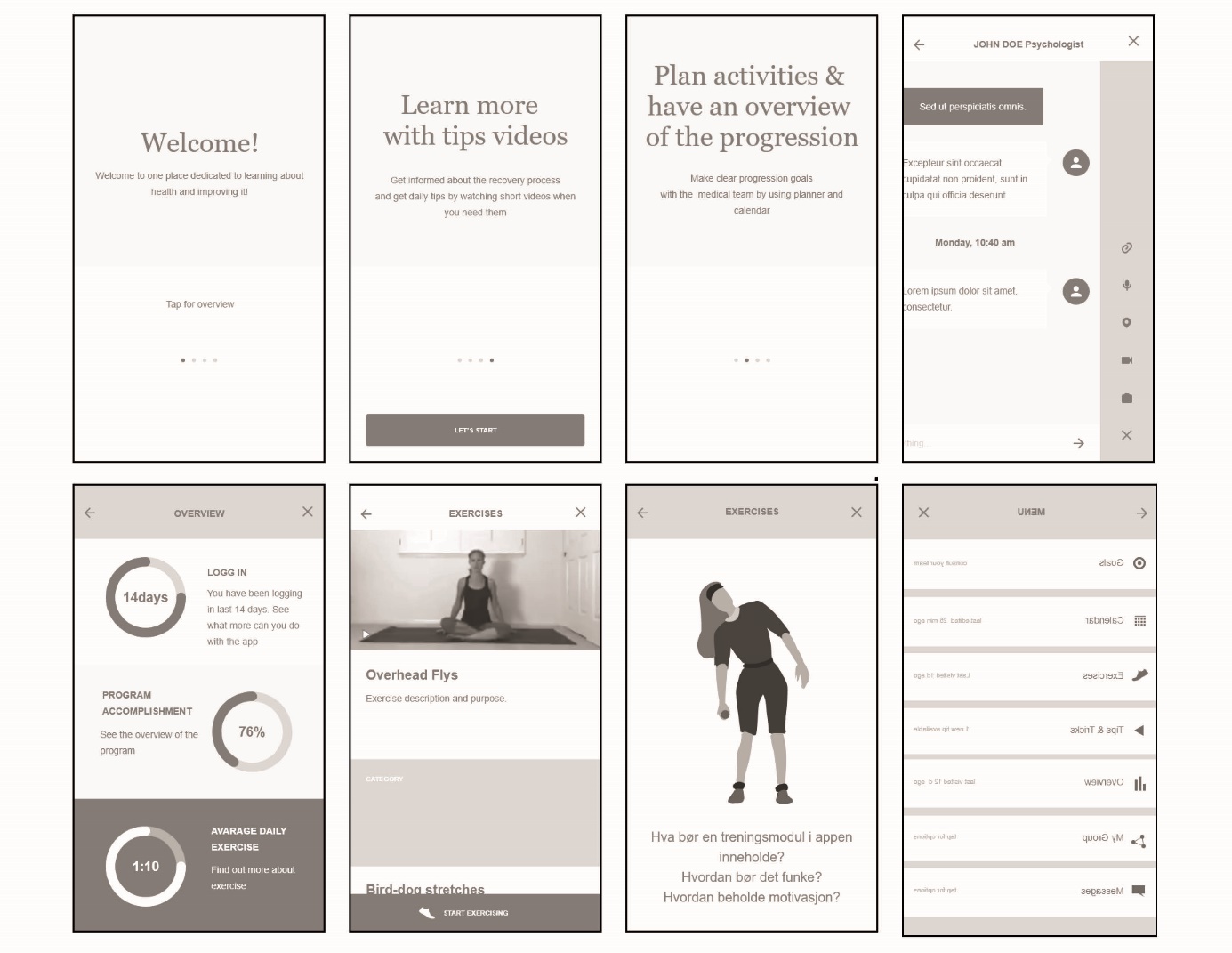

Supplement: Multimedia Appendix 3 [file jmir_v27i1e69089_app3.docx]
