# Supplementary material for: Exploring Stakeholders’ Perceptions of Using Digital Health Technologies to Improve the Conservative Treatment of Adolescent Idiopathic Scoliosis: Qualitative Study
Source: J Med Internet Res. 2025 Jun 25;27:e69089. doi: 10.2196/69089 (PMC12242061; doi:10.2196/69089)

**Multimedia Appendix 4.** Version of the prototype presented at workshop number 5. The prototype was modified based on feedback received in the previous workshop.


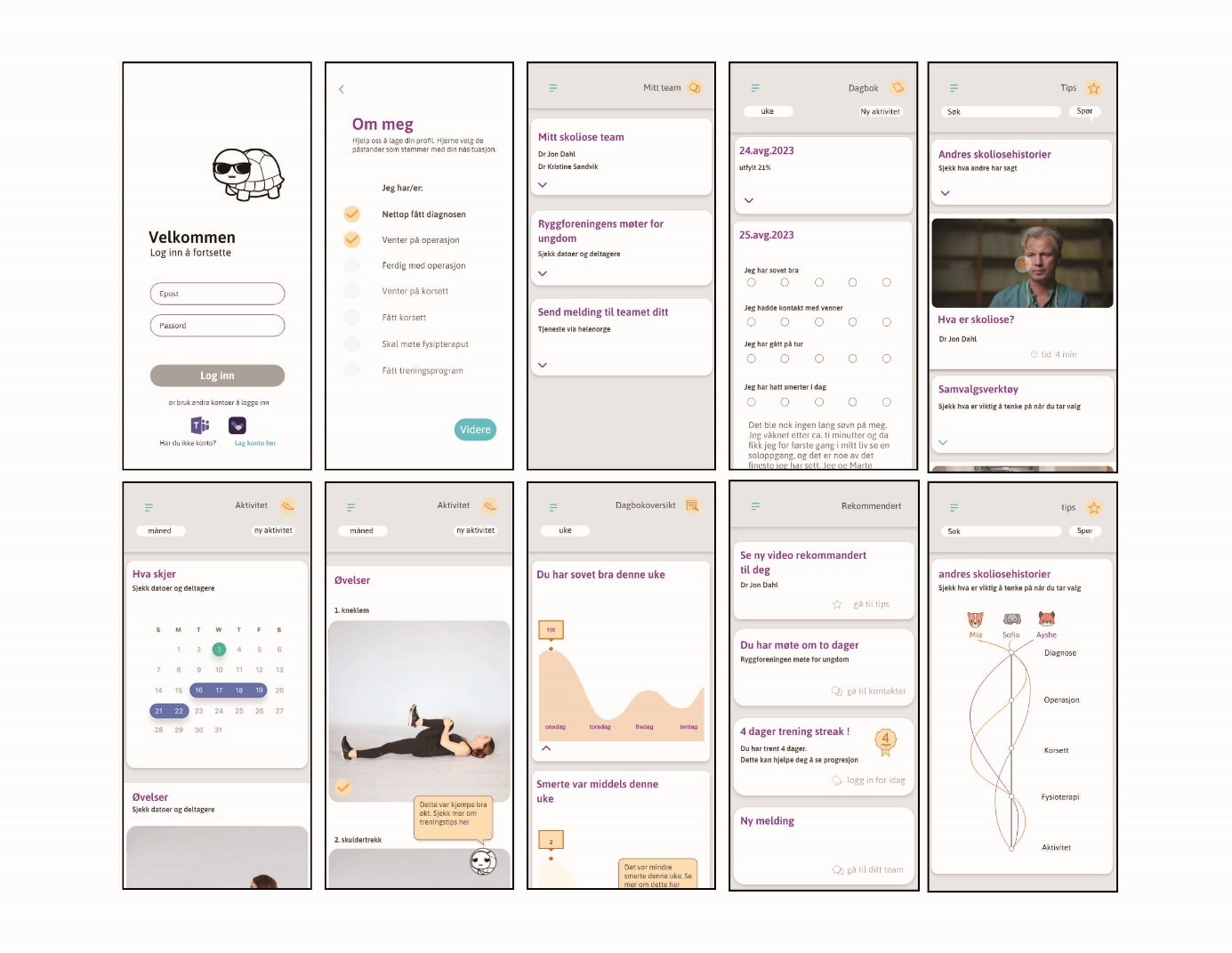

Supplement: Multimedia Appendix 4 [file jmir_v27i1e69089_app4.docx]
